# Deficiencies in Natura 2000 for protecting recovering large carnivores: A spotlight on the wolf *Canis lupus* in Poland

**DOI:** 10.1371/journal.pone.0184144

**Published:** 2017-09-05

**Authors:** Tom A. Diserens, Tomasz Borowik, Sabina Nowak, Maciej Szewczyk, Natalia Niedźwiecka, Robert W. Mysłajek

**Affiliations:** 1 Institute of Genetics and Biotechnology, Faculty of Biology, University of Warsaw, Warsaw, Poland; 2 Mammal Research Institute, Polish Academy of Sciences, Białowieża, Poland; 3 Association for Nature “Wolf”, Twardorzeczka, Poland; Università degli Studi di Napoli Federico II, ITALY

## Abstract

If protected areas are to remain relevant in our dynamic world they must be adapted to changes in species ranges. In the EU one of the most notable such changes is the recent recovery of large carnivores, which are protected by Natura 2000 at the national and population levels. However, the Natura 2000 network was designed prior to their recent recovery, which raises the question whether the network is sufficient to protect the contemporary ranges of large carnivores. To investigate this question we evaluated Natura 2000 coverage of the three wolf *Canis lupus* populations in Poland. Wolf tracking data showed that wolves have recolonised almost all suitable habitat in Poland (as determined by a recent habitat suitability model), so we calculated the overlap between the Natura 2000 network and all wolf habitat in Poland. On the basis of published Natura 2000 criteria, we used 20% as the minimum required coverage. At the national level, wolves are sufficiently protected (22% coverage), but at the population level, the Baltic and Carpathian populations are far better protected (28 and 47%, respectively) than the endangered Central European Lowland population (12%). As Natura 2000 insufficiently protects the most endangered wolf population in Poland, we recommend expansion of Natura 2000 to protect at least an additional 8% of wolf habitat in western Poland, and discuss which specific forests are most in need of additional coverage. Implementation of these actions will have positive conservation implications and help Poland to fulfil its Habitats Directive obligations. As it is likely that similar gaps in Natura 2000 are arising in other EU member states experiencing large carnivore recoveries, particularly in Central Europe, we make the case for a flexible approach to Natura 2000 and suggest that such coverage evaluations may be beneficial elsewhere.

## Introduction

Protected areas are some of the most effective strategies for conserving endangered species and their habitats [1]. Among them, the EU’s flagship biodiversity programme Natura 2000 is the largest internationally coordinated network of protected areas [2]. Natura 2000 has been systematically evaluated several times for various taxa [3–5], and numerous studies have shown that overlap between priority areas and Natura 2000 sites is low (so-called ‘gaps’) [6–10], and that the network is inadequate for ensuring long-term persistence of many European protected species [11, 12].

One of the primary ways such gaps can arise is through the use of deficient species range data during Natura 2000 planning stages [13, 14]. But little attention has been given to gaps arising over time due to changes in species ranges—either due to species expansion, contraction or range shift (e.g. due to climate change). This is surprising as we live in a dynamic world, where on the one hand, anthropogenic pressures and climate changes may drive decreases in wildlife distributions, or on the other, conservation activities may allow endangered species to recover and increase their ranges. Thus it could be expected that the Habitats Directive would stipulate a mechanism for routinely filling in gaps in Natura 2000 arising from such processes; however, no such mechanism exists, as demonstrated by the lack of significant changes to the Natura 2000 network since it was established. Thus it appears that Natura 2000 is inflexible and incapable of keeping up with changes in a dynamic world. This is problematic, as without regular modifications the Natura 2000 network will over time acquire increasing numbers of gaps that will gradually erode its relevancy as a conservation instrument.

The recovery of large carnivores in Europe is one of few recent conservation success stories [15], driving some of the largest recent changes in species ranges. The Habitats Directive and Natura 2000 have played a key role in protecting large carnivores; however, the Natura 2000 network across Europe was designed prior to their recent recovery. Thus the question arises whether the existing network is sufficient to protect the contemporary ranges of large carnivores. To investigate this question we evaluated Natura 2000 coverage of the wolf *Canis lupus*, Linnaeus 1758 in Poland, a country that has been experiencing a significant expansion of the wolf over the last decade. Our aim was to find out whether the Polish Natura 2000 network has been adapted to cover areas recently recolonised by the wolf, in accordance with the requirements of the Habitats Directive. As large carnivores are predicted to further increase their ranges and numbers in Europe, particularly in Central Europe, this knowledge is important to determine whether large carnivores are being sufficiently protected in recently recolonised areas.

### The wolf and Natura 2000 in Poland

In Poland, as elsewhere in Europe, the wolf suffered centuries of persecution that led to considerable range contractions. By the 1990s the wolf was mostly confined to the northeast and southeast of the country, and was absent west of the Vistula [16]. Over time however, increasing scientific knowledge and environmental consciousness led to the strict protection of the species in 1998 [17]. The wolf population has since been recovering, and by 2012 had recolonised most large forests in western Poland [18]. Wolves now number ca. 1,500 individuals in Poland [19], the majority of which inhabit the Carpathian Mountains and vast forests in the east of the country [20], with western Poland still in the process of the being recolonised [18, 21, 22].

Poland’s accession to the EU in 2004 brought further protection for the wolf in the form of the Habitats Directive (Directive 92/43/EEC) [23]. In Poland the directive protects the wolf on annexes II − needing habitat conservation, and V − where the taking in the wild and exploitation is subject to management restrictions [24].

To assist with the transboundary nature and conflict-prone characteristics of large carnivores, the European Commission developed management guidelines for their conservation (referred to as the LC guidelines hereinafter) [24]. These represent the best available science and experience for managing European large carnivore populations. The LC guidelines indicate that member states should maintain species at FCS at the population level, but with the caveat that ‘many (maybe most) countries will never be able to host enough individuals to have a population that can reach FCS’ [24]. In such cases member states are obliged to meaningfully contribute towards maintaining FCS of the populations partially within their borders [25, 26]. Eventually, Transboundary working groups should be set up to coordinate management objectives across national borders—but these do not yet exist for any Polish wolf population [27].

Despite the apparently clear advice for member states to manage species at the population level, there remains some confusion about appropriate level at which to conduct management efforts. This confusion arises when viewing recent European Court of Justice (ECJ) jurisprudence. In separate cases brought against Sweden and Finland, the ECJ assessed the conservation status of the wolf at the national levels, despite these countries sharing their wolf populations with neighbouring countries (Finland with Russia, and Sweden with Norway). In both cases the member states did not have the necessary degree of transboundary cooperation set out in the LC guidelines. In an attempt to make sense of these judgements Trouwborst et al. [26] suggested that ‘until a fully-fledged and well-functioning transboundary plan is in place (…) the national level would be the default scale for assessing FCS for large carnivore populations’. The contradictory conclusions of the LC guidelines and ECJ jurisprudence lead to an interesting conclusion: until Poland establishes transboundary management plans for its three populations, it must contribute towards maintaining the wolf at FCS at both the national, and population levels.

To aid population level management, the authors of the LC guidelines delimitated populations of large carnivores across Europe. In Poland they identified three wolf populations: the Baltic, Carpathian, and Central European Lowland (CE hereinafter) populations. These populations are at varying degrees of recovery (Table 1), where the Carpathian and Baltic are at FCS, while the CE is not [15] and is listed as endangered on the IUCN red list [28].

**Table 1:** Population sizes and red list statuses for the three wolf populations partially within Poland. Population figures for Poland are from Kaczensky et al. [29] and transboundary figures and red list statuses are from Boitani et al. [28].

Population	Population size in Poland	Transboundary population size (within EU)	Red list status
**Baltic**	267–359	~900–1,400	Least concern
**Carpathian**	209–254	~3,500	Least concern
**CE**	100–110	300	Endangered

The Habitats Directive requires member states protect large carnivore populations within national Natura 2000 networks. To this end, special areas of conservation (SACs) are designated to protect areas of habitat essential to a species’ life and reproduction [23] (referred to as ‘core areas’ of species habitat hereinafter). This requirement to protect species’ core areas can be difficult to interpret as there is no comprehensive guidance document for Natura 2000 implementation, and there is little published information from the Natura 2000 planning period [30]. Thus criteria by which to assess Natura 2000 network sufficiency are obscure, and evaluating coverage of large carnivores can be a complex task.

In particular, two important questions arise when assessing coverage of populations. Firstly, what are species’ core areas of habitat? This concept by definition must vary from species to species depending on species-specific ecological requirements. But there is, to our knowledge, no discussion about this within the literature. Secondly, how much of a population should Natura 2000 protect? This was a crucial question during the Natura 2000 planning stages, so the EC published criteria for the assessment of member state proposals and their subsequent approval [31]. These have been described as the ‘20–60% guidelines’ [30]–where for most species between 20 and 60% of a population should be protected within Natura 2000. These criteria are not prescriptive, and instead advise that coverage levels should be determined on a case-by-case basis depending on species ecology, distribution and diversity, and population trends and abundance [30]. A scientist who worked in Natura 2000 planning confirmed to us that the 20–60% guidelines were also used in Poland, where below 20% coverage of a population was invariably too low, and 60% was usually plenty (P. Pawlaczyk 2016, pers. comm., 4 November). However, how the concepts of core areas and coverage relate to the wolf remain unpublished, and surprisingly, there is little mention of Natura 2000 within the LC guidelines.

Poland created its first SACs after joining the EU in 2004. Preparatory work began in the late 1990s, but due to delays in implementation, the completed list of 823 SACs was finalised in 2010 [32]. After a few site additions and expansions, Poland now has 849 SACs covering an area of 38,525.97 km^2^. However, as elsewhere in Europe, Natura 2000 in Poland pre-dates the late 2000s recovery of the wolf [18], so may be inadequate for protecting the current, more extensive wolf range. Most likely to have insufficient coverage is the endangered CE population, which was severely restricted 15 years ago, and is now recovering [18]. If coverage is insufficient, the Habitats Directive obliges member states to extend Natura 2000 to cover recently recolonised habitats.

To investigate the flexibility of Natura 2000 to respond to changes in species’ ranges we evaluated the Natura 2000 coverage of the wolf at both the national and population levels in Poland. We identified which areas have the least coverage and thus which forests should be targeted during future Natura 2000 network expansions. This is the first study to evaluate the population level Natura 2000 coverage of a European large carnivore, and implementation of the actions we recommend will have several positive conservation implications and help Poland to fulfil its Habitats Directive obligations. Our findings suggest similar gaps may exist or may be arising in other EU member states experiencing large carnivore recoveries; thus we make the case for a flexible approach to Natura 2000 and suggest that such coverage evaluations may be beneficial elsewhere.

## Methods

### Study area

Poland’s area (about 311,900 km^2^; 49°80'00''–54°85'00''N, 14°80'80''– 24°80'90''E) is mainly plains, with 91% of the country < 300 m above mean sea level. Glaciations have shaped the lowland landscape (mainly the Riss, 310,000–130,000 years ago, and the Würm, 70,000–10,000 years ago). Major mountain ranges (the Sudety and Carpathian Mts.) span the southern national borders. Forests cover 29% of the country. Agricultural land covers about 60%, dominated by arable fields, and with smaller amounts of meadows, pastures, and orchards. Mean human population density is 123 individuals/km^2^ [33].

Poland is located in the temperate climate zone, transitional between Atlantic and continental types. Mean temperature in January ranges from 0 to 11°C on the Baltic coast and in western Poland, to -5.5°C in the north-east and -7°C in the mountains. Mean temperature in July ranges from 10°C in the mountains to 16.5°C at the sea and 19°C in south-west. Mean annual precipitation averages from 500–650 mm in the lowlands to 1200–1500 mm in the mountains, and snow cover persists for 60–100 days.

### Habitat suitability model

We evaluated the Natura 2000 coverage of all wolf habitat in Poland irrespective of wolf recolonisation status. Coverage evaluations usually evaluate coverage of the current range of a species (e.g. [2, 34–36]); however, the wolf is quickly recolonising the remaining vacant habitat in Poland [18], and if current trends continue, all suitable habitat in Poland will soon host wolves. We chose this approach instead of evaluating coverage of the current wolf range to ensure the study will remain future-proof, and not become obsolete as the wolf recolonises more areas.

As a reference for wolf habitat in Poland we used a recent habitat suitability model [37], which has proven to be a good predictor of wolf recolonisation over the past decade [22]. This model was built by dividing the area of Poland into 10×10 km cells characterized in terms of their habitat variables: percentage area covered by forests, wetlands and marshes, meadows and pastures, arable fields, settlements and buildings, as well their density of major roads and crude biomass of wild ungulates. Next, data on wolf occurrence in eastern Poland collected over 7 years (2000–2006) were used to parameterize a resource selection function relating wolf occurrence to the habitat variables. The variables were then tested for collinearity to determine the most parsimonious set of variables, with the following pairs of variables being the most highly correlated: forests and arable fields, forests and ungulate biomass, settlements and buildings and roads. From each pair, only the more relevant variable for wolf habitat preference or avoidance was selected for further analysis (in these cases, forests and roads—see Jędrzejewski et al. [38, 39]). The remaining variables, percentage cover of forests, meadows and marshes (positively correlated to wolf abundance) and road density (negatively correlated), were then used in a multivariate model to predict probabilities of wolf occurrence in cells across Poland. Finally, cells were grouped into 33 habitat patches, the natural borders of which were delimitated by mapping their contiguous forests and wetlands/marshes.

Despite their importance for predicting wolf occurrence and tailoring management [40], food resources (ungulate biomass) were not considered a separate variable in the model predicting probabilities of wolf occurrence. This is because in Poland forest cover is a proxy for food resources due to the ubiquitous presence of ungulates in forests across the country, as shown by the most recent comprehensive ungulate census [41]. Furthermore, the most recent government statistics (2016) show that since this census ungulate population numbers in Poland have been rising [19]. Thus all patches in Poland have abundant food resources, which do not play a key role in wolf management.

The 33 patches comprise all areas of suitable wolf habitat > 400 km^2^ in Poland, with an additional five < 400 km^2^ patches included to cover ‘the whole range of variation in wolf numbers and physiographic conditions [in Poland]’. All patches are contiguous tracts of forests and wetlands/marshes, altogether covering 61,592km^2^ and 19.75% of the country. Out of 34 patches, 18 are small (<1,000 km^2^), 10 are medium-sized (1,000–4,000 km^2^) and 5 are very large (> 4,000 km^2^). They range in size from 160 to 8,338 km^2^. The Baltic and CE populations comprise several patches of a range of different sizes. In contrast, the Carpathian population has a smaller range within Poland and comprises 3 patches, one very large and two medium sized.

The elevations of patches range from sea level at the Baltic coast, up to > 2,000 m at the tops of highest mountain ridges in the south. Patches are mainly characterized by mixed temperate forest, largely in the form of commercial stands managed by the State Forestry but also include forests in national parks and small nature reserves. Dominant tree species are Scots pine (*Pinus sylvestris*), Norway spruce (*Picea abies*), beech (*Fagus sylvatica*), and fir (*Abies alba*), with the latter 2 species occurring mainly in the mountains. Other trees include oak (*Quercus robur*), ash (*Fraxinus excelsior*), birch (*Betula pubescens* and *B*. *verrucosa*), hornbeam (*Carpinus betulus*), maple (*Acer platanoides*), and black alder (*Alnus glutinosa*).

### Software and datasets

We carried out GIS analysis in ArcMap v.10.2.2. SHP layers for SACs in Poland were obtained from the General Directorate for Environmental Protection website [42], and for all wolf habitat in Poland from the habitat suitability model in Jędrzejewski et al. [37]. Statistical analyses were carried out in SPSS v.23 (Mann-Whitney U tests), and R (Pearson’s Chi-squared test and Fisher’s exact tests) [43].

### GIS analysis

**Fig 1:**
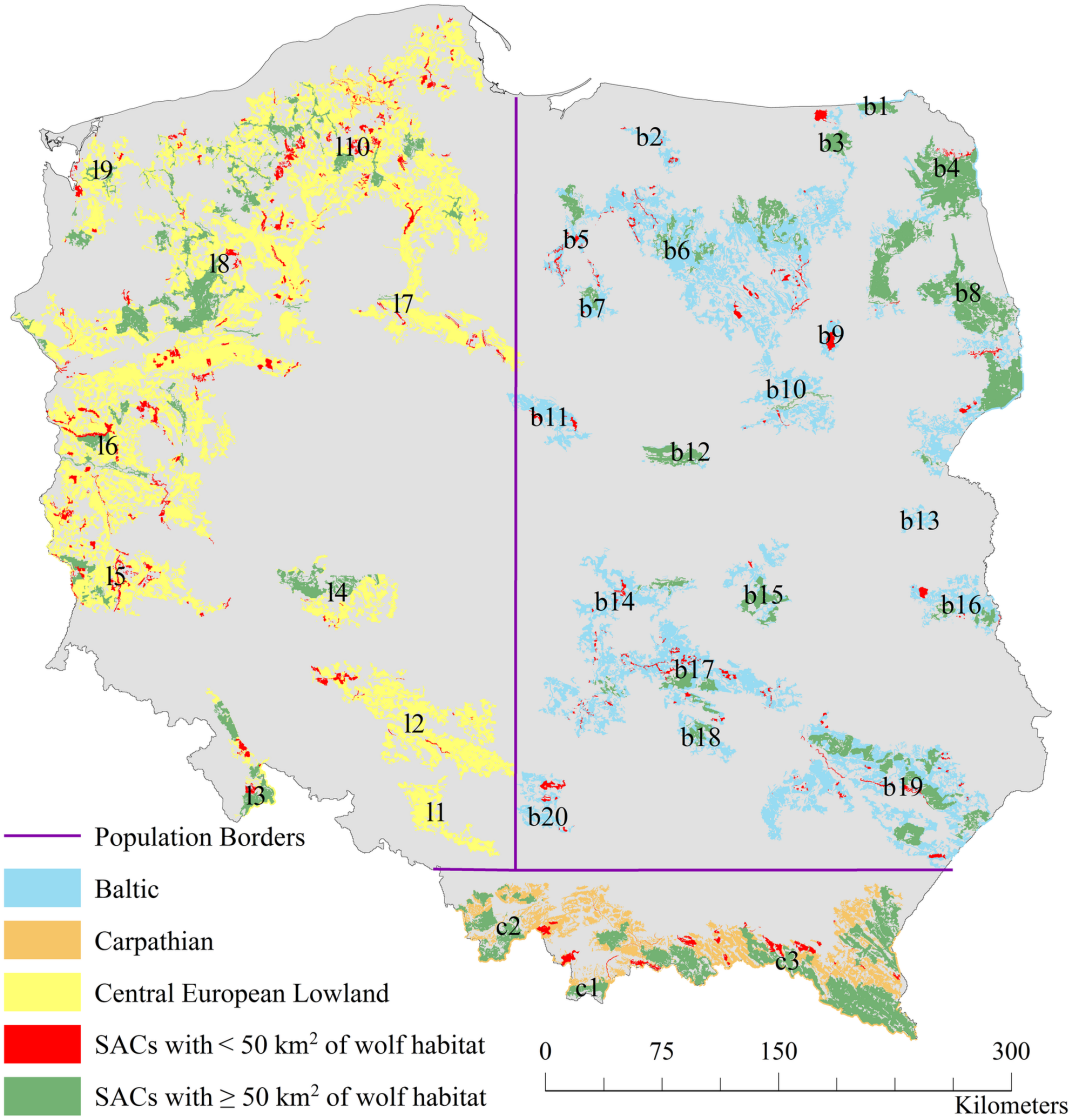
Suitable habitat patches for wolves in Poland. Habitat belonging to populations is shown in blue, orange and yellow. The protected habitat is shown in green and red, denoting areas protected by sites with ≥ 50 km^2^ of wolf habitat, and areas protected by sites with < 50 km^2^ of wolf habitat, respectively. Patch labels correspond to those used later in the text.

We overlaid the wolf habitat and SAC layers in ArcMap (Fig 1), and then calculated the area of overlap of every SAC with wolf habitat patch polygons, using the calculate intersection tool. This gave information on the amount (area in km^2^) of wolf habitat protected by each SAC. In our analysis we only included sites protecting ≥ 1 km^2^ of wolf habitat and we grouped them into two categories:

1. Sites protecting ≥ 50 km^2^ of wolf habitat. This threshold was based on the average size of denning and rendezvous sites (core-territories) of breeding female wolves in Poland (49 km^2^) [44] and corresponds to the Habitats Directive requirement to protect species’ ‘core areas’ of habitat.
2. Sites protecting < 50 km^2^ of wolf habitat—sites that protect areas of wolf habitat smaller than the core area size. These were not included in the coverage evaluation as the amount of wolf habitat they protect is too small to play a major role in wolf conservation. However, they are potential targets for network expansion.

#### Wolf occurrence

We collected data on permanent wolf occurrence at each patch and Natura 2000 site to confirm our assumption that wolves inhabit almost all wolf habitat in Poland. Wolf occurrence data was obtained from recent literature [15, 18] and wolf monitoring of the Conservation Genetics Group, Institute of Genetics and Biotechnology, Faculty of Biology, University of Warsaw and Association for Nature “Wolf” − wolf monitoring data were obtained by tracking, camera trapping, DNA analysis and telemetry. The wolf occurrence data was then added to the wolf habitat and SAC SHP databases.

#### Populations

For the population level analysis, we divided the study area into three geographic regions (Fig 1). For this we adapted the borders of Linnell et al. [24], to reflect the most up to date knowledge on the genetic structuring of Polish wolves [45]: the Carpathian border was moved southwards, and the Baltic-CE border westwards. We then visually inspected the habitat map (Fig 1) and tagged SACs and habitat patches according to which population they belong. Patches and SACs that straddled population borders belonged to a particular population if > 50% of their areas fell into a population’s range.

#### Protected wolf habitat statistics

**Fig 2:**
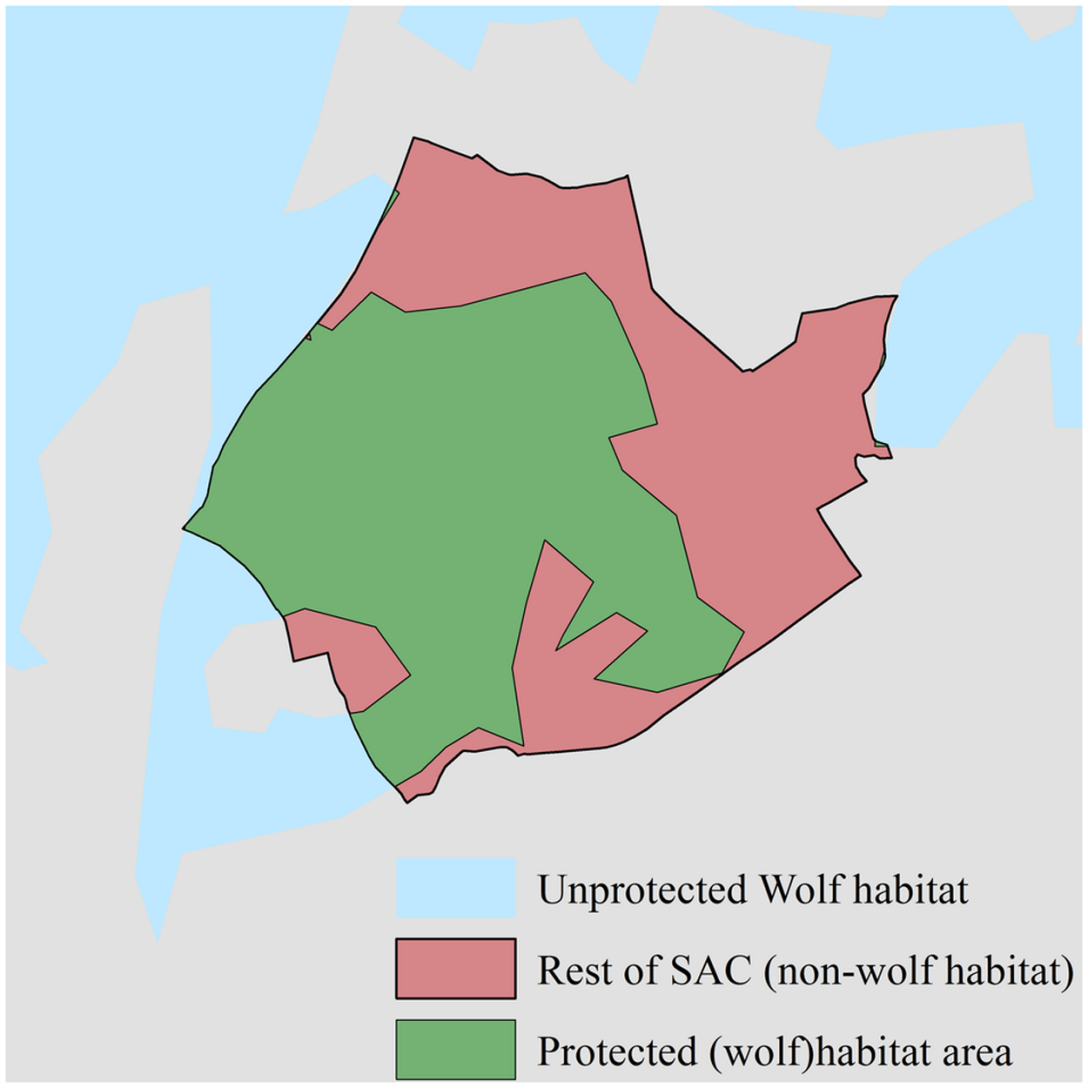
Visualisation of a protected habitat area. Protected wolf habitat is shown in green and is differentiated from the rest of the SAC (red), which protects non-wolf habitat. Unprotected wolf habitat outside the borders of the SAC is shown in blue. The rest of the landscape (non-wolf habitat, non-SAC) is shown in grey.

We calculated and then compared the average sizes of wolf habitat protected by SACs in each of the three populations. We first differentiated the wolf habitat portions of SACs apart from the non-wolf habitat (see Fig 2 for visualisation of this concept). We used this method because comparing overall site sizes between populations would have led to erroneous results as it would have included areas of non-wolf habitat. These differentiated areas of wolf habitat were termed **protected habitat areas**. We calculated the average size and standard deviation of protected habitat areas ≥ 50km^2^ in size for each of the populations in excel. The sizes of protected habitat areas in each of the populations were then compared (pairwise) using Mann-Whitney U tests. Differences were considered significant when P < 0.05.

### Coverage evaluation

#### Minimum coverage threshold

In the absence of legal clarity or comprehensive guidance document for establishing terrestrial Natura 2000 sites, we generated our own threshold for the minimum coverage level. The 20–60% guidelines suggest that coverages below 20% may be suitable for species that are ‘widespread, extensive and show a limited range of ecological or genetic variation’ [31]. However, the wolf in Poland has only recently been recovering from large historical range contractions, and the endangered CE population is still not widespread or extensive [15, 18]. Furthermore, Polish wolves have considerable genetic structuring, which may reflect ecological differences between the populations [45]. These factors suggest that sub 20% coverage levels would not be permissible for the wolf in Poland. For this reason, we decided that Natura 2000 should protect at least 20% of a population’s range, based on the lower limit in the 20–60% guidelines. Furthermore, this coverage should include only sites with ≥ 50 km^2^ of wolf habitat, accounting for the requirement to protect wolf core areas.

#### National level coverage

We evaluated Natura 2000 coverage at the national level (i.e. of all wolf habitat in Poland) by calculating the percentage overlap between SACs and habitat patch polygons using the calculate intersection tool in ArcMap. The percentage overlap was then compared with the 20% coverage threshold.

#### Population level coverage

Next we evaluated Natura 2000 coverage of each population. The patches were grouped according to the population to which they belong. As figures for the area protected within each patch were obtained during the national level coverage analysis, the total amount of habitat protected in each population was determined by adding up the protected habitat within that population. Percentage coverages were then calculated by dividing the total protected population habitat by the total population habitat. These coverage figures were then judged against the 20% coverage threshold. Coverages were compared using a Pearson Chi-square test. Pairwise comparisons between each of the populations were then conducted with Fisher’s exact test.

## Results

### Wolf occurrence

Wolves permanently occur in 26/33 habitat patches in Poland, thus confirming our assumption that most habitat in Poland has already been recolonised by the wolf. All of Poland’s large and all but one medium patch host wolves. Out of the seven patches not hosting wolves, 6 are small and one is medium-sized.

### Protected habitat area comparison

There are 295 SACs/protected habitat areas in Poland protecting ≥ 1 km^2^ of wolf habitat (S1 Table), 68 of which protect ≥ 50 km^2^ of wolf habitat (Table 2). Considering only the protected habitat areas ≥ 50 km^2^ in size, the Baltic population has the fewest and on average the largest protected habitat areas, whereas the CE population has the most and on average the smallest protected habitat areas, which are half the size of the country-wide average, and almost a third of the size of those within the Baltic population. There were significant differences in the sizes of protected habitat areas between the CE population and both of the other two populations. In contrast, there was no difference between the Baltic and Carpathian populations; Mann-Whitney U-Tests (P values can be found in S2 Table).

**Table 2:** Number of sites and average protected habitat area sizes for each population. Table only includes protected habitat areas ≥ 50 km^2^.

Population	No. Sites	Average protected habitat area size (km^2^)
Baltic	16	333 ± 314
Carpathian	23	219 ± 199
CE	29	126 ± 121
Poland	68	206 ± 223

### Coverage evaluation

The evaluation of wolf habitat coverage only includes sites that protect ≥ 50 km^2^ of wolf habitat, sites protecting smaller areas of habitat were excluded for being too small to protect wolf core areas.

#### Coverage at the national level

With 22% of all wolf habitat in Poland protected within Natura 2000 (Table 3), the national population is sufficiently protected (minimum 20%). However, the distribution of protected habitat is uneven, and biased towards the east and south of the country (visible in Fig 1, where extensive green shaded areas are located in the east and south).

**Table 3:** Wolf habitat patches and populations in Poland and their Natura 2000 coverages. Total coverage figures for each population are shown at the ends of each section, and for the national level are shown at the end of the table. Only sites that protect ≥ 50 km^2^ of wolf habitat were included in the calculations. Patch labels are as in Fig 1.

Patch label	Patch name	Patch size (km^2^)	Wolf presence at patch	Area protected by Natura 2000 (km^2^)	Natura 2000 percentage coverage
**b1**	Romnicka Forest	160	✔	105	66
**b2**	Dobre Miasto-Orneta	208	✔	0	0
**b3**	Borki Forest	328	✔	174	53
**b4**	Augustów Forest-Biebrza River Valley	2139	✔	1,765	83
**b5**	Iława Forest	512	✖	152	30
**b6**	Napiwoda-Ramuki-Pisz Forest	4,788	✔	648	14
**b7**	Górzno-Lidzbark Landscape Park	331	✔	73	22
**b8**	Knyszyn-Białowieża-Mielnik Forest	2,694	✔	1,692	63
**b9**	Czerwony Bór	173	✔	0	0
**b10**	Biała Forest	885	✔	47	5
**b11**	Włocławek-Gostynin Forest	519	✔	0	0
**b12**	Kampinos Forest	304	✔	278	91
**b13**	Międzyrzec Podlaski-Biała Podlaska	150	✔	0	0
**b14**	Pilica Forest	790	✔	86	11
**b15**	Kozienice Forest	639	✖	260	41
**b16**	Parczew-Sobibór Forest	740	✔	136	18
**b17**	Włoszczowa-Opoczno-Swięty Krzyż-Iłża Forest	2,886	✔	287	10
**b18**	Cisowsko-Orłowiński Landscape Park	351	✖	95	27
**b19**	Roztocze-Sandomierz-Solska Forest	3,868	✔	689	18
**b20**	Kraków-Częstochowa Upland	438	✖	0	0
	**Total for Baltic Population**	**22,906**	-	**6,487**	**28**
**c1**	Carpathians I (Tatra Mts)	354	✔	134	38
**c2**	Carpathians II (Żywiec-Silesian Beskids)	1,501	✔	584	39
**c3**	Carpathians III (Gorce-Bieszczady Mountains-Przemyśl Foothills)	5,511	✔	2,752	50
	**Total for Carpathian Population**	**7,365**	-	**3,470**	**47**
**l1**	Racibórz Forest	720	✖	0	0
**l2**	Silesian Forest	2,650	✖	0	0
**l3**	Eastern and Central Sudetes	621	✔	351	57
**l4**	Barycz Forest	1,111	✖	392	35
**l5**	Lower Silesian Forest	1,995	✔	188	9
**l6**	NW Poland II	7,152	✔	337	5
**l7**	Bydgoszcz Forest	1547	✔	13	1
**l8**	NW Poland I	6,139	✔	1313	21
**l9**	Bukowa-Goleniów Forest	1,050	✔	174	17
**l10**	NW Poland III	8,338	✔	894	11
	**Total for CE population**	**31,321**	**-**	**3,662**	**12**
	**Total for all of Poland**	**61,592**	**-**	**13,619**	**22**

#### Coverage at the population level

Coverages of the three wolf populations vary significantly (χ^2^ = 5161, P < 0.001). The Carpathian population (47% of habitat area protected within SACs) has almost double the coverage of the Baltic population (28%), which in turn has over double the coverage of the CE population (12%) (Table 3). Thus, Natura 2000 sufficiently protects the Baltic and Carpathian populations (above the 20% threshold), but under-protects the CE population by almost 50%. Pairwise comparisons with Fisher’s exact test indicated that all population coverages are different from each other (all comparisons: p < 0.001).

#### Baltic population

The Baltic population has sufficient coverage (Table 3). But patches are covered somewhat unevenly: four patches have 0% and another four have over 50% coverage. Two of the largest tracts of wolf habitat are very well protected by Natura 2000 (Augustów Forest-Biebrza River Valley and Knyszyn- Białowieża-Mielnik Forest patches), and correspondingly, protecting them are several large Natura 2000 sites containing large amounts of wolf habitat (including Białowieża, Augustów, Knyszyn and Biebrza SACs–S1 Table). Furthermore, wolves have not yet recolonised four patches within the range of this population (Iława Forest, Kozienice Forest, Cisowsko-Orłowiński Landscape Park, and Krakow-Czestochowa Upland); three of these already have good coverage (Iława– 30%, Kozienice– 40%, and Cisowsko-Orłowiński Landscape Park– 27%). Overall this population is well protected

#### Carpathian population

The Carpathian population has the most extensive coverage (Table 3). Each of the three Carpathian patches host wolves, and each has almost double the minimum coverage requirement. Several large SACs cover each of the patches (see S1 Table for list of sites). The largest patch the Carpathian III patch is the best protected, with 50% coverage. Overall this population is very well protected, and so are each of its three patches.

#### Central European Lowland population

Coverage of this population is both insufficient and erratic (Table 3). All but three small patches host wolves, and their coverages range from ≤5% (in Racibórz, Silesian, and Bydgoszcz Forests, and NW Poland II) to 35% in Barycz Forest, and even 57% in the Eastern and Central Sudetes, with few patches of intermediate coverage levels. Notably, the two largest patches, NW Poland II and NW Poland III, comprising almost a half of the total suitable habitat for this population, have just 5 and 11% coverage, respectively. Overall this endangered wolf population is thoroughly underprotected.

### Additional protection

Each population also has a number of SACs that protect < 50 km^2^ of wolf habitat (Table 4). These sites protect too little wolf habitat to protect wolf core areas but may protect wolf habitat generally. Notably, the CE population has the largest number of sites protecting areas of wolf habitat too small to be meaningful for wolf conservation. The sites identified in this analysis are prime targets for expansion—for a list of sites, see S1 Table.

**Table 4:** Number of sites protecting < 50 km^2^ of wolf habitat in each population. A list of all Natura 2000 sites protecting wolf habitat can be found in S1 Table.

Population	Number of sites
Baltic	34
Carpathian	56
CE	137
Whole of Poland	227

## Discussion

Our results shown how in Poland a recovering large carnivore, the wolf, has insufficient Natura 2000 coverage within the recently recolonised parts of its range. We found that coverage is sufficient at the national level, and for the Baltic and Carpathian populations, but not for the recovering, endangered CE population. This is the first study to evaluate Natura 2000 coverage in recently recolonised areas of a European protected species’ range, and the first to evaluate the population level coverage of a large carnivore. Previous studies have focussed on coverage at the national or continental levels (e.g. [36]) despite the fact that the Habitats Directive requires member states contribute to maintaining species at FCS at both the national and population levels simultaneously.

The novel population level aspect of the study, and two assumptions in our methodology make comparisons with the literature difficult: namely, i) the requirement for a minimum of 20% coverage of wolf habitat, and ii) the interpretation of core areas as ≥ 50 km^2^ of wolf habitat, corresponding to the size of breeding female wolf denning and rendezvous sites. Natura 2000 assessors interpret these subjectively depending on the species in question, and their own interpretations of Habitats Directive requirements. However, in the absence of guidelines on protecting large carnivores within Natura 2000 and prior ECJ jurisprudence, these concepts will continue to be interpreted in various ways, and for the reasons already mentioned we believe our interpretations are the best suited to the requirements of the wolf in Poland. In light of the lack of suitable comparable data in the literature, we discuss our results with reference to relevant published data only where this is possible.

### Coverage at the national level

In Poland, Natura 2000 provides sufficient coverage (22%) for the wolf at the national level. During the Natura 2000 network planning phases, SACs were designated with reference to the early 2000s wolf range. But at this time wolves were restricted mostly to the east and south of the country [20]; consequently, most large protected habitat areas are in the east and south. Since then, wolves have been recolonising new, unprotected habitat, causing coverage to drop over time. Nevertheless, coverage at the national level will remain sufficient even as wolves fill up the last available habitat. Increasing coverage for the wolf at the national level in Poland is therefore not a priority. However, fine-scale analysis of the network to determine whether SACs overlap with areas currently being utilised as wolf core areas may be beneficial (discussed in more detail in the recommendations section below).

### Coverage at the population level

The Carpathian and Baltic populations—which span areas that already hosted many wolves during the Natura 2000 planning stages—have more (and sufficient) coverage and on average larger protected habitat areas than the endangered CE population, which has mostly been recovering over the last 10 years [18]. Statistical comparisons of protected habitat area sizes between the populations suggest that CE protected habitat areas are different from those in the Baltic and Carpathian populations. A discussion on the reasoning for these differences now follows.

#### Baltic & Carpathian populations

The Baltic and Carpathian populations have sufficient coverage and similarly sized protected habitat areas for two main reasons. Firstly, sizeable populations of the wolf and lynx lived here in the early 2000s (during the Natura 2000 planning phase) [20], so many sites in eastern and southern Poland were designed especially for large carnivores. Secondly, in eastern and southern Poland there are several large areas with high levels of biodiversity, most of which are prime wolf habitat (i.e. ranked as very good wolf habitat in the habitat suitability model). These include the Białowieża, Augustów and Pisz Forests, Biebrza Marshes, and Carpathians, some of which have been described as biodiversity hotspots [46, 47]. Thus, the large SACs designated primarily to protect the diversity of species in these areas also provide coverage for wolves. Alternatively, this could be viewed the other way around: Natura 2000 planners had an incentive to provide extensive coverage for the wolf (and large carnivores in general) as this protects overall biodiversity through the umbrella effect [48]. For these reasons, in these populations, even as the wolf has recolonised new areas, coverage has remained sufficient − the network was future-proof.

One notable difference between the Baltic and Carpathian populations is in their total coverages: the Baltic population has around half the coverage of the Carpathian. This is probably because the Baltic has by comparison roughly three times more wolf habitat than the Carpathian population, so reaching the same levels of coverage would require the protection of vast more tracts of forest (several thousands of km^2^).

Coverage levels for these populations will remain sufficient even as the last habitat vacant habitats are recolonised, especially as there are several uncolonised patches within the Baltic population that already have good Natura 2000 coverage. Natura 2000 network expansion here is therefore not a priority; however, as for the national level, fine-scale analysis of SACs to determine their overlap with areas currently being utilised as wolf core areas would be beneficial.

#### Central European Lowland population

In contrast, coverage of this endangered population is insufficient. Protected habitat area sizes are also significantly different, and on average smaller than in the other two populations. Furthermore, the CE population has a large number (~3x more than the Carpathian, and ~6x more than Baltic) of sites protecting areas of wolf habitat too small (< 50 km^2^) to be meaningful for wolf conservation. This situation is somewhat surprising as ‘species coverage generally increases with threat level, as based on IUCN red list status’ [49]: but in Poland, the most endangered wolf population has the lowest coverage. This is because during the Natura 2000 network planning phases wolf recolonisation of western Poland was in its early stages [18, 20]. As there weren’t many wolves to cover at this time, coverage of wolf habitat is low, and protected habitat area sizes are different because SACs were designed for species other than the wolf, which have different conservation requirements. Ultimately, Natura 2000 network planners did not anticipate wolf recolonisation, and so did not make the network future-proof. Wolves have since recolonised all but three CE patches, including the three largest patches in Poland. In light of the recent recolonization of unprotected habitat, Natura 2000 should be extended to cover at least an additional 8% of wolf habitat in western Poland (up from the current 12% to 20%). Also, as above, fine-scale analysis of SACs would be beneficial. Specific actions on how to implement both of these actions can be found in the recommendations section below.

### General remarks

Polish wolf habitat gains some additional protection from sites that protect < 50 km^2^ of wolf habitat. These sites protect areas of wolf habitat too small to fulfil the Habitats Directive’s obligations to protect wolf core areas, but may improve the quality of wolf habitat generally (e.g. by protecting wolf prey species), and may function as stepping stones in the dispersal process [36]. These sites are a missed opportunity to provide additional coverage to wolf core areas, as many sites require only small expansions to protect sufficient amounts (50 km^2^) of wolf habitat. Where network expansions are necessary, rather than create new sites, it may be easier to expand the borders of these existing sites to overlap with wolf core areas.

In the coverage evaluation we included all SACs with ≥ 50km^2^ of wolf habitat and did not consider the shapes and locations of SACs: these are important factors for the effectiveness of protected areas for the conservation of wolves [50, 51]. Some sites in our evaluation could therefore be either unsuitably shaped (e.g. a site protecting a river valley) or suboptimally located (e.g. adjacent to a settlement). However, a study looking into the shaping and location of each of the 68 SACs that protect ≥ 50 km^2^ of wolf habitat in Poland would be time consuming, and would have to include field data on where wolves in each area do, or are likely to locate their core areas. This was outside the scope of this study, but as a consequence of these unknown factors, coverage levels in this study may be somewhat overestimated, depending on the number of included irregularly shaped or suboptimally located SACs. In light of this we recommend future fine-scale analysis of the Polish Natura 2000 network—more details can be found in the recommendations section below.

There are two contrasting points of note regarding our use of total wolf habitat. On the one hand, contrary to many independent assessments (e.g. [2, 34–36]), we evaluated coverage of all wolf habitat in the country irrespective of wolf recolonisation status, as opposed to coverage of the current range of the species. This is a potential criticism, as expecting coverage in areas of the country that have not yet been recolonised is perhaps unrealistic. However, using a current range approach for the wolf in Poland would have quickly led to outdated results as the wolf is rapidly recolonising the remaining vacant habitat in the country [18]. Thus, in order for our results to be future-proof, we evaluated coverage of the maximum potential wolf range in Poland (as per the habitat suitability model). On the other hand, there is a medium-long term opposing consequence of using this approach. As wolves fill up good wolf habitat, they may be pushed into increasingly suboptimal habitat, i.e. closer to human settlements and the margins of forests—areas that the habitat suitability model may not have captured as suitable wolf habitat [22, 52]. As a result, even the optimistic scenario used in this study may eventually become inadequate. But this is perhaps a scenario that was unanticipated during the creation of the Habitats Directive, and there is a future debate to be made as to whether wolves are desired and/or should be protected outside of natural/semi-natural habitats once populations are at FCS.

### Recommendations

We recommend Poland carry out two actions for fulfilment of its Habitats Directive obligations. The first should be carried out for all populations in Poland. The second and highest priority action concerns the insufficiently covered and endangered CE population.

#### 1) Fine-scale analysis of SACs

To be effective and to meet legal requirements, Natura 2000 sites should overlap with wolf core areas. A fine-scale analysis of the Polish Natura 2000 network, on a site-by-site basis, would determine the level of overlap between SACs and actual wolf core areas. Such an analysis will require good quality field data about areas that wolves are actively utilising as denning and rendezvous sites (i.e. wolf core areas). Polish SACs could then be optimised: their borders moved so that they more suitably cover wolf core areas. This would increase protection of wolf packs, without increasing Natura 2000 network extent.

#### 2) Natura 20000 expansion

**Fig 3:**
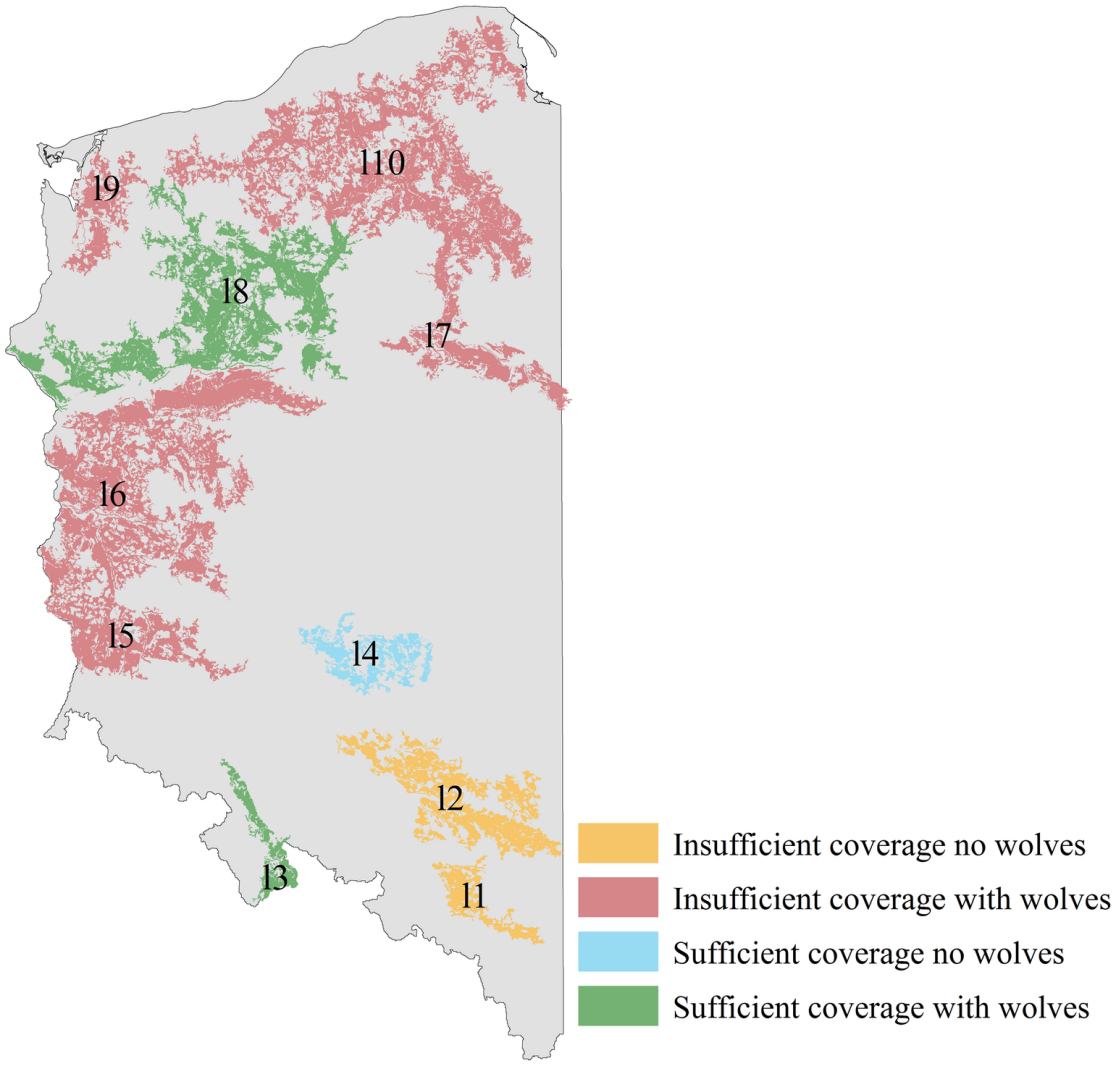
Sufficiency of coverage and wolf recolonisation status of CE population patches. The five red patches with < 20% coverage that host wolves are the highest priority for Natura 2000 expansion. Labels are as in Table 3.

Expanding coverage of the endangered CE population should be done by creating new, or expanding existing SACs. Fig 3 shows the patches most in need of additional coverage. The priority patches for network expansion are those with < 20% coverage that already host wolves (red patches in Fig 3); a further two patches should be protected as and when they are recolonised by wolves (orange patches in Fig 3). Polish authorities should consider the following points when increasing coverage:

1. SACs should protect the best quality wolf habitat. Thus network expansions should overlap with habitat ranked as very good (≥ 50% chance of hosting wolves) in the habitat suitability model.
2. Good quality field data should be used to identify current wolf core areas. SACs can then be designated to overlap with them.
3. Expansion of existing sites that already host wolves should be prioritised over the creation of brand new sites, especially those protecting < 50 km^2^ of wolf habitat (32 such sites exist in western Poland–S1 Table).
4. New coverage should be distributed between several patches, particularly those with the least coverage to ensure an even distribution of coverage across the country.

### Implications

In addition to merely fulfilling legal obligations, expanding Natura 2000 coverage to recently recolonised areas of a species’ range will have positive conservation implications. Natura 2000 can play a key role in achieving, and then maintaining FCS of wolf populations. This is despite protected areas having some limitations for the protection of large carnivores, and the fact that conservation at Natura 2000 sites is often ‘limited to just publishing regulations limiting human activity’ [36]. There is evidence suggesting that even relatively small reserves (smaller than a large carnivore home range) can function as refuges in landscapes with no official protection status, allowing large carnivores to avoid the most dangerous aspects of close proximity to humans [53]. This is particularly important in western Poland, which comprises some of the most densely populated and economically developed provinces in the country [33]. Rapid economic growth will likely continue for the foreseeable future, bringing increased anthropogenic pressures, such as infrastructure development, that threaten biodiversity: these are threats that Natura 2000 can effectively mitigate despite its current limitations. Furthermore, the recent ‘nature directive fitness check’ [54] prompted the EC to develop the 2017 European Action Plan for nature, people and the economy [55]. This plan gives solutions to the current issues with active management, enforcement, and limited resources at Natura 2000 sites and is to be implemented over the coming years. Thus, in time, Natura 2000 has the potential to grow into an even more valuable instrument for the conservation of the wolf and other large carnivores in Europe.

Natura 2000 expansion in western Poland will also benefit species other than the wolf. In the absence of comprehensive coverage assessments for any other European protected species in Poland, there could be further undetected gaps in the network, especially if species have undergone recent range expansion, e.g. Eurasian lynx *Lynx lynx* [56] and European bison *Bison bonasus* [57]. In these cases, the wolf’s role as an ‘umbrella species’ may help moderate the impact of such gaps by capturing them within the network [35, 48], and therefore improve general protection of biodiversity in western Poland.

### Future research directions

Similar gaps in Natura 2000 are likely to be arising in other EU member states experiencing large carnivore recoveries. Thus it would be beneficial for these member states to carry out similar evaluations as in the present study. Indeed, the CE wolf population is now recolonising Germany, Czechia and even Denmark and the Netherlands [45, 58, 59], and there are suggestions that the German and Czech portions of this population’s range have similar coverage issues [27, 36]. Coverage evaluations and corresponding Natura 2000 expansions in member states bordering Poland will not only help meet national conservation and legal requirements, but would also contribute towards management at the transboundary level. Future transboundary management plans will have to consider each member states’ contributions to the maintenance of each population at FCS at the transboundary level. Thus good quality coverage data will allow working groups to draw up coordinated actions to modify Natura 2000 so that the wolf can be optimally managed irrespective international borders.

More broadly, the European Commission should note that there is currently no effective mechanism for Natura 2000 to keep up with changes in species’ ranges. Over time, this limitation may lead to increasing gaps in the Natura 2000 network as species change their distributions in response to anthropogenic pressures, conservation measures, and climate change. A possible solution would be to integrate Natura 2000 coverage evaluations and modifications into the Habitats Directive’s article 7 monitoring and reporting of conservation statuses procedure. Introducing such a flexible approach would enable routine filling of gaps, allowing Natura 2000 to maintain its relevance into the coming decades.

## Conclusion

Our results show how the recovery of a large carnivore creates gaps in the Natura 2000 network. We propose that Natura 2000 must have a flexible approach to address these gaps—it must be adapted on the basis of up to date, good quality species’ range data. We have recommended two actions to fill the identified gaps and effectively protect the wolf within natura 2000 in Poland: firstly, fine-scale analysis of SACs should be carried out for all Polish wolf populations to determine the extent of SAC coverage of actual wolf core areas, and secondly, the Natura 2000 network should be expanded in western Poland to cover an additional 8% of the CE population. Implementation of the recommended actions will have several positive conservation implications and help Poland to fulfil its Habitats Directive obligations. It is likely that similar gaps in Natura 2000 are arising in other EU member states experiencing large carnivore recoveries: thus similar evaluations as in the present study may be beneficial elsewhere.

## Supporting information

S1 Table List and details of all SACs in Poland with ≥ 1 km^2^ of wolf habitat. Sites are sorted by population, and then by the amount of wolf habitat they protect.
(DOCX)

S2 Table Statistical significance of the Mann-Whitney U-tests for the pairwise comparisons of protected habitat area sizes between wolf populations in Poland. (DOCX)
