# Supplementary material for: Deficiencies in Natura 2000 for protecting recovering large carnivores: A spotlight on the wolf Canis lupus in Poland
Source: PLoS One. 2017 Sep 5;12(9):e0184144. doi: 10.1371/journal.pone.0184144 (PMC5584752; doi:10.1371/journal.pone.0184144)
Supplement: S1 Table — Sites are sorted by population, and then by the amount of wolf habitat they protect. (DOCX) [file pone.0184144.s001.docx]

**S1 Table. List and details of all SACs in Poland with ≥ 1 km^2^ of wolf habitat.** Sites are sorted by population, and then by the amount of wolf habitat they protect.

| Site Code | Population | Wolf Presence | Site Area (km^2^) | Wolf Habitat Area (km^2^) | Wolf Habitat Percentage Area (%) |
| --- | --- | --- | --- | --- | --- |
| PLH200006 | Baltic | ✔ | 1361 | 1069 | 79 |
| PLH200005 | Baltic | ✔ | 1071 | 937 | 88 |
| PLH200008 | Baltic | ✔ | 1212 | 713 | 59 |
| PLC200004 | Baltic | ✔ | 631 | 597 | 95 |
| PLH280048 | Baltic | ✔ | 578 | 422 | 73 |
| PLC140001 | Baltic | ✔ | 376 | 278 | 74 |
| PLH140035 | Baltic | ✖ | 282 | 260 | 92 |
| PLH280052 | Baltic | ✔ | 326 | 226 | 69 |
| PLH280016 | Baltic | ✔ | 253 | 174 | 69 |
| PLH280053 | Baltic | ✖ | 210 | 152 | 72 |
| PLH200004 | Baltic | ✔ | 161 | 114 | 71 |
| PLH280005 | Baltic | ✔ | 148 | 105 | 71 |
| PLH060043 | Baltic | ✔ | 97 | 82 | 85 |
| PLH140011 | Baltic | ✔ | 460 | 74 | 16 |
| PLH280012 | Baltic | ✔ | 89 | 73 | 82 |
| PLH060013 | Baltic | ✔ | 102 | 54 | 53 |
| PLH200018 | Baltic | ✔ | 51 | 48 | 95 |
| PLH280049 | Baltic | ✔ | 114 | 47 | 41 |
| PLH280001 | Baltic | ✖ | 126 | 32 | 25 |
| PLH060107 | Baltic | ✔ | 36 | 31 | 87 |
| PLH200010 | Baltic | ✔ | 191 | 27 | 14 |
| PLH200007 | Baltic | ✔ | 136 | 25 | 18 |
| PLH040036 | Baltic | ✖ | 42 | 23 | 54 |
| PLH140052 | Baltic | ✖ | 22 | 22 | 99 |
| PLH200019 | Baltic | ✔ | 25 | 22 | 87 |
| PLH280006 | Baltic | ✔ | 84 | 19 | 23 |
| PLH140049 | Baltic | ✖ | 19 | 19 | 97 |
| PLH200024 | Baltic | ✖ | 186 | 17 | 9 |
| PLH140047 | Baltic | ✖ | 11 | 11 | 98 |
| PLH200023 | Baltic | ✖ | 32 | 11 | 34 |
| PLH280036 | Baltic | ✖ | 14 | 11 | 74 |
| PLH200020 | Baltic | ✖ | 14 | 11 | 73 |
| PLH280046 | Baltic | ✖ | 12 | 10 | 88 |
| PLH280015 | Baltic | ✖ | 13 | 8 | 67 |
| PLH280035 | Baltic | ✖ | 9 | 8 | 85 |
| PLH140032 | Baltic | ✖ | 136 | 6 | 5 |
| PLH140030 | Baltic | ✖ | 15 | 5 | 36 |
| PLH140046 | Baltic | ✖ | 6 | 5 | 94 |
| PLH280003 | Baltic | ✖ | 8 | 5 | 64 |
| PLH280030 | Baltic | ✖ | 6 | 5 | 73 |
| PLH280033 | Baltic | ✖ | 15 | 4 | 25 |
| PLH060011 | Baltic | ✖ | 5 | 3 | 54 |
| PLH060032 | Baltic | ✖ | 82 | 3 | 3 |
| PLH140013 | Baltic | ✖ | 3 | 3 | 84 |
| PLH280040 | Baltic | ✖ | 3 | 2 | 90 |
| PLH280039 | Baltic | ✖ | 2 | 2 | 76 |
| PLH040035 | Baltic | ✖ | 2 | 2 | 87 |
| PLH280054 | Baltic | ✖ | 16 | 1 | 9 |
| PLH280056 | Baltic | ✖ | 13 | 1 | 9 |
| PLH140008 | Baltic | ✖ | 1 | 1 | 90 |
| PLC180001 | Carpathian | ✔ | 1115 | 989 | 89 |
| PLH120019 | Carpathian | ✔ | 579 | 471 | 81 |
| PLH180013 | Carpathian | ✔ | 461 | 380 | 82 |
| PLH060034 | Carpathian | ✔ | 347 | 315 | 91 |
| PLH180012 | Carpathian | ✔ | 397 | 308 | 78 |
| PLH060031 | Carpathian | ✔ | 345 | 306 | 89 |
| PLH240006 | Carpathian | ✔ | 353 | 277 | 78 |
| PLH180014 | Carpathian | ✔ | 293 | 241 | 83 |
| PLH240005 | Carpathian | ✔ | 264 | 239 | 90 |
| PLH180001 | Carpathian | ✔ | 201 | 193 | 96 |
| PLH180054 | Carpathian | ✔ | 180 | 174 | 97 |
| PLH120018 | Carpathian | ✔ | 180 | 170 | 94 |
| PLH260010 | Carpathian | ✔ | 191 | 164 | 85 |
| PLC120001 | Carpathian | ✔ | 210 | 134 | 64 |
| PLH260040 | Carpathian | ✖ | 104 | 95 | 91 |
| PLH140016 | Carpathian | ✖ | 318 | 86 | 27 |
| PLH060017 | Carpathian | ✔ | 85 | 82 | 97 |
| PLH260031 | Carpathian | ✖ | 78 | 77 | 98 |
| PLH180017 | Carpathian | ✔ | 116 | 76 | 66 |
| PLH260002 | Carpathian | ✖ | 81 | 74 | 91 |
| PLH240023 | Carpathian | ✔ | 72 | 68 | 95 |
| PLH260004 | Carpathian | ✖ | 116 | 68 | 58 |
| PLH060093 | Carpathian | ✔ | 58 | 50 | 86 |
| PLH240009 | Carpathian | ✖ | 58 | 48 | 82 |
| PLH120016 | Carpathian | ✔ | 83 | 45 | 54 |
| PLH180016 | Carpathian | ✖ | 52 | 43 | 81 |
| PLH260015 | Carpathian | ✔ | 58 | 36 | 63 |
| PLH060097 | Carpathian | ✔ | 85 | 35 | 41 |
| PLH120001 | Carpathian | ✔ | 34 | 33 | 98 |
| PLH120036 | Carpathian | ✖ | 33 | 27 | 83 |
| PLH260018 | Carpathian | ✖ | 112 | 26 | 23 |
| PLH180015 | Carpathian | ✖ | 27 | 26 | 93 |
| PLH120094 | Carpathian | ✖ | 28 | 25 | 87 |
| PLH260038 | Carpathian | ✖ | 23 | 23 | 96 |
| PLH180055 | Carpathian | ✔ | 80 | 22 | 27 |
| PLH180018 | Carpathian | ✖ | 23 | 21 | 93 |
| PLH100008 | Carpathian | ✖ | 38 | 21 | 56 |
| PLH180024 | Carpathian | ✖ | 23 | 21 | 92 |
| PLH260011 | Carpathian | ✔ | 24 | 20 | 84 |
| PLC120002 | Carpathian | ✔ | 23 | 20 | 86 |
| PLH100003 | Carpathian | ✔ | 20 | 17 | 85 |
| PLH180020 | Carpathian | ✖ | 102 | 15 | 15 |
| PLH260025 | Carpathian | ✖ | 15 | 15 | 96 |
| PLH260028 | Carpathian | ✖ | 36 | 13 | 35 |
| PLH120025 | Carpathian | ✖ | 19 | 12 | 66 |
| PLH120014 | Carpathian | ✖ | 20 | 11 | 57 |
| PLH260001 | Carpathian | ✖ | 24 | 10 | 43 |
| PLH160008 | Carpathian | ✖ | 11 | 10 | 91 |
| PLH120035 | Carpathian | ✖ | 20 | 10 | 50 |
| PLH060083 | Carpathian | ✖ | 9 | 9 | 98 |
| PLH260019 | Carpathian | ✖ | 26 | 9 | 34 |
| PLH180026 | Carpathian | ✔ | 12 | 8 | 72 |
| PLH120012 | Carpathian | ✔ | 8 | 8 | 100 |
| PLH060094 | Carpathian | ✔ | 11 | 7 | 64 |
| PLH260026 | Carpathian | ✖ | 8 | 7 | 85 |
| PLH260024 | Carpathian | ✖ | 7 | 7 | 97 |
| PLH180021 | Carpathian | ✖ | 16 | 7 | 42 |
| PLH120024 | Carpathian | ✖ | 7 | 6 | 90 |
| PLH120005 | Carpathian | ✖ | 9 | 6 | 64 |
| PLH180028 | Carpathian | ✖ | 6 | 6 | 98 |
| PLH180048 | Carpathian | ✔ | 5 | 4 | 84 |
| PLH120088 | Carpathian | ✖ | 8 | 4 | 56 |
| PLH120033 | Carpathian | ✖ | 13 | 4 | 31 |
| PLH060028 | Carpathian | ✖ | 4 | 4 | 99 |
| PLH100035 | Carpathian | ✖ | 5 | 3 | 72 |
| PLH260012 | Carpathian | ✖ | 8 | 3 | 44 |
| PLH100024 | Carpathian | ✖ | 3 | 3 | 100 |
| PLH160010 | Carpathian | ✖ | 8 | 3 | 34 |
| PLH180052 | Carpathian | ✖ | 27 | 2 | 9 |
| PLH120037 | Carpathian | ✖ | 6 | 2 | 41 |
| PLH240027 | Carpathian | ✖ | 2 | 2 | 99 |
| PLH120006 | Carpathian | ✖ | 6 | 2 | 40 |
| PLH260035 | Carpathian | ✖ | 2 | 2 | 99 |
| PLH100026 | Carpathian | ✖ | 2 | 2 | 100 |
| PLH060022 | Carpathian | ✖ | 2 | 2 | 100 |
| PLH060003 | Carpathian | ✖ | 2 | 2 | 100 |
| PLH060089 | Carpathian | ✖ | 2 | 2 | 96 |
| PLH120093 | Carpathian | ✖ | 2 | 1 | 55 |
| PLH180034 | Carpathian | ✖ | 2 | 1 | 58 |
| PLH320046 | CE | ✔ | 744 | 653 | 88 |
| PLH020041 | CE | ✖ | 820 | 392 | 48 |
| PLH080071 | CE | ✔ | 266 | 246 | 92 |
| PLH020016 | CE | ✖ | 190 | 164 | 86 |
| PLH320007 | CE | ✖ | 277 | 161 | 58 |
| PLH320039 | CE | ✖ | 319 | 148 | 46 |
| PLH320022 | CE | ✖ | 219 | 148 | 68 |
| PLH020071 | CE | ✖ | 213 | 132 | 62 |
| PLH080044 | CE | ✔ | 122 | 120 | 98 |
| PLH320023 | CE | ✔ | 150 | 105 | 70 |
| PLH220034 | CE | ✖ | 136 | 102 | 75 |
| PLH080037 | CE | ✔ | 112 | 102 | 91 |
| PLH220078 | CE | ✔ | 100 | 90 | 90 |
| PLH320020 | CE | ✖ | 120 | 90 | 75 |
| PLH220038 | CE | ✖ | 143 | 86 | 60 |
| PLH080002 | CE | ✖ | 153 | 85 | 55 |
| PLH080028 | CE | ✖ | 192 | 82 | 43 |
| PLH320013 | CE | ✖ | 84 | 75 | 90 |
| PLH320067 | CE | ✔ | 102 | 74 | 72 |
| PLH320044 | CE | ✖ | 88 | 68 | 78 |
| PLH020072 | CE | ✔ | 81 | 68 | 84 |
| PLH220026 | CE | ✔ | 75 | 68 | 90 |
| PLH320049 | CE | ✖ | 148 | 65 | 44 |
| PLH080008 | CE | ✔ | 68 | 60 | 89 |
| PLH320003 | CE | ✖ | 83 | 59 | 71 |
| PLH320037 | CE | ✔ | 305 | 57 | 19 |
| PLH020096 | CE | ✖ | 71 | 56 | 78 |
| PLH040017 | CE | ✔ | 63 | 55 | 88 |
| PLH300004 | CE | ✖ | 505 | 51 | 10 |
| PLH080011 | CE | ✔ | 50 | 46 | 91 |
| PLH320009 | CE | ✖ | 65 | 43 | 66 |
| PLH320001 | CE | ✖ | 48 | 42 | 88 |
| PLH160009 | CE | ✖ | 44 | 41 | 93 |
| PLH040023 | CE | ✔ | 39 | 38 | 97 |
| PLH080060 | CE | ✔ | 44 | 38 | 86 |
| PLH220052 | CE | ✖ | 70 | 38 | 54 |
| PLH020050 | CE | ✔ | 60 | 37 | 62 |
| PLH080003 | CE | ✖ | 74 | 35 | 47 |
| PLH080001 | CE | ✖ | 71 | 33 | 46 |
| PLH020062 | CE | ✖ | 34 | 33 | 96 |
| PLH020017 | CE | ✖ | 88 | 32 | 36 |
| PLH080014 | CE | ✖ | 60 | 30 | 50 |
| PLH300045 | CE | ✖ | 31 | 29 | 94 |
| PLH300032 | CE | ✖ | 76 | 28 | 37 |
| PLH020063 | CE | ✔ | 101 | 27 | 27 |
| PLH220057 | CE | ✖ | 38 | 27 | 72 |
| PLH220079 | CE | ✖ | 65 | 26 | 41 |
| PLH080036 | CE | ✔ | 30 | 26 | 86 |
| PLH320033 | CE | ✖ | 27 | 25 | 90 |
| PLH320040 | CE | ✖ | 34 | 24 | 72 |
| PLH320045 | CE | ✖ | 66 | 24 | 37 |
| PLH220036 | CE | ✖ | 55 | 23 | 42 |
| PLH080032 | CE | ✔ | 23 | 23 | 100 |
| PLH020019 | CE | ✖ | 54 | 23 | 42 |
| PLH300037 | CE | ✖ | 23 | 23 | 99 |
| PLH320048 | CE | ✔ | 32 | 22 | 69 |
| PLH080009 | CE | ✖ | 22 | 22 | 99 |
| PLH220077 | CE | ✖ | 25 | 20 | 82 |
| PLH220098 | CE | ✖ | 23 | 20 | 86 |
| PLC080001 | CE | ✖ | 333 | 19 | 6 |
| PLH320025 | CE | ✔ | 22 | 19 | 85 |
| PLH020015 | CE | ✔ | 67 | 18 | 27 |
| PLH220006 | CE | ✖ | 26 | 18 | 71 |
| PLH320012 | CE | ✖ | 26 | 18 | 67 |
| PLH320006 | CE | ✖ | 208 | 17 | 8 |
| PLH080055 | CE | ✔ | 18 | 16 | 93 |
| PLH080042 | CE | ✔ | 16 | 16 | 100 |
| PLH080012 | CE | ✖ | 31 | 16 | 53 |
| PLH080015 | CE | ✖ | 20 | 16 | 82 |
| PLH040029 | CE | ✖ | 28 | 16 | 56 |
| PLH140021 | CE | ✖ | 16 | 16 | 97 |
| PLH300017 | CE | ✔ | 18 | 15 | 86 |
| PLH220014 | CE | ✖ | 16 | 15 | 95 |
| PLH040012 | CE | ✔ | 39 | 15 | 38 |
| PLH080069 | CE | ✖ | 15 | 15 | 97 |
| PLH040031 | CE | ✖ | 39 | 14 | 36 |
| PLH080068 | CE | ✖ | 17 | 14 | 79 |
| PLH040041 | CE | ✔ | 53 | 13 | 25 |
| PLH020086 | CE | ✖ | 24 | 13 | 54 |
| PLH220005 | CE | ✖ | 25 | 12 | 49 |
| PLH080070 | CE | ✖ | 12 | 12 | 97 |
| PLH080007 | CE | ✔ | 14 | 12 | 85 |
| PLH320018 | CE | ✖ | 526 | 12 | 2 |
| PLH220062 | CE | ✖ | 17 | 12 | 69 |
| PLH320038 | CE | ✖ | 15 | 11 | 78 |
| PLH080067 | CE | ✖ | 13 | 11 | 85 |
| PLH320011 | CE | ✖ | 20 | 11 | 55 |
| PLH220058 | CE | ✖ | 15 | 11 | 74 |
| PLH220041 | CE | ✖ | 14 | 11 | 77 |
| PLH300043 | CE | ✖ | 14 | 10 | 70 |
| PLH300006 | CE | ✔ | 10 | 10 | 94 |
| PLH080031 | CE | ✖ | 9 | 9 | 100 |
| PLH220027 | CE | ✖ | 9 | 9 | 96 |
| PLH020018 | CE | ✖ | 202 | 9 | 4 |
| PLH300003 | CE | ✖ | 9 | 9 | 96 |
| PLH320052 | CE | ✖ | 8 | 8 | 93 |
| PLH300046 | CE | ✖ | 8 | 8 | 98 |
| PLH300031 | CE | ✖ | 8 | 7 | 83 |
| PLH080057 | CE | ✖ | 7 | 7 | 95 |
| PLH040011 | CE | ✖ | 14 | 7 | 49 |
| PLH320061 | CE | ✖ | 9 | 7 | 75 |
| PLH220080 | CE | ✖ | 9 | 7 | 75 |
| PLH080046 | CE | ✖ | 10 | 6 | 63 |
| PLH080063 | CE | ✖ | 6 | 6 | 100 |
| PLH080048 | CE | ✖ | 6 | 6 | 100 |
| PLH080033 | CE | ✖ | 6 | 6 | 97 |
| PLH080039 | CE | ✖ | 6 | 6 | 99 |
| PLH220056 | CE | ✖ | 8 | 6 | 72 |
| PLH320056 | CE | ✖ | 6 | 6 | 98 |
| PLH300047 | CE | ✖ | 9 | 6 | 62 |
| PLH020090 | CE | ✔ | 6 | 6 | 100 |
| PLH320065 | CE | ✖ | 6 | 5 | 95 |
| PLH300021 | CE | ✔ | 22 | 5 | 24 |
| PLH220061 | CE | ✖ | 5 | 5 | 99 |
| PLH320002 | CE | ✖ | 6 | 5 | 82 |
| PLH020052 | CE | ✖ | 8 | 5 | 55 |
| PLH300042 | CE | ✖ | 5 | 4 | 84 |
| PLH020078 | CE | ✖ | 21 | 4 | 20 |
| PLH220094 | CE | ✖ | 46 | 4 | 9 |
| PLH020091 | CE | ✖ | 11 | 4 | 38 |
| PLH220029 | CE | ✖ | 8 | 4 | 54 |
| PLH220016 | CE | ✖ | 4 | 4 | 98 |
| PLH160012 | CE | ✖ | 9 | 4 | 44 |
| PLH320036 | CE | ✖ | 8 | 4 | 52 |
| PLH080052 | CE | ✖ | 8 | 4 | 49 |
| PLH220064 | CE | ✔ | 5 | 4 | 83 |
| PLH320066 | CE | ✖ | 5 | 4 | 80 |
| PLH040022 | CE | ✖ | 5 | 4 | 78 |
| PLH320053 | CE | ✖ | 5 | 4 | 83 |
| PLH040039 | CE | ✖ | 48 | 4 | 8 |
| PLH220099 | CE | ✖ | 4 | 3 | 97 |
| PLH300016 | CE | ✖ | 5 | 3 | 73 |
| PLH080064 | CE | ✖ | 4 | 3 | 89 |
| PLH220060 | CE | ✖ | 3 | 3 | 100 |
| PLH080038 | CE | ✖ | 4 | 3 | 73 |
| PLH080004 | CE | ✖ | 5 | 3 | 64 |
| PLH020043 | CE | ✖ | 3 | 3 | 91 |
| PLH220037 | CE | ✖ | 10 | 3 | 27 |
| PLH220081 | CE | ✖ | 4 | 3 | 72 |
| PLH220020 | CE | ✖ | 3 | 3 | 100 |
| PLH220066 | CE | ✖ | 3 | 2 | 69 |
| PLH080049 | CE | ✖ | 3 | 2 | 75 |
| PLH220012 | CE | ✖ | 2 | 2 | 100 |
| PLH020007 | CE | ✖ | 2 | 2 | 99 |
| PLH220028 | CE | ✖ | 2 | 2 | 96 |
| PLH080013 | CE | ✖ | 8 | 2 | 20 |
| PLH220096 | CE | ✖ | 11 | 2 | 15 |
| PLH320008 | CE | ✖ | 2 | 2 | 100 |
| PLH320004 | CE | ✖ | 45 | 2 | 4 |
| PLH020093 | CE | ✖ | 14 | 2 | 11 |
| PLH020092 | CE | ✖ | 2 | 2 | 98 |
| PLH040026 | CE | ✖ | 11 | 2 | 14 |
| PLH080073 | CE | ✖ | 3 | 1 | 49 |
| PLH220071 | CE | ✖ | 4 | 1 | 40 |
| PLH080059 | CE | ✖ | 2 | 1 | 90 |
| PLH300019 | CE | ✖ | 2 | 1 | 60 |
| PLH220070 | CE | ✖ | 4 | 1 | 33 |
| PLH160013 | CE | ✖ | 4 | 1 | 38 |
| PLH220039 | CE | ✖ | 1 | 1 | 100 |
| PLH320010 | CE | ✖ | 2 | 1 | 74 |
| PLH140051 | CE | ✖ | 1 | 1 | 99 |
| PLH320069 | CE | ✖ | 15 | 1 | 9 |
| PLH220019 | CE | ✖ | 3 | 1 | 45 |
| PLH080053 | CE | ✖ | 2 | 1 | 54 |
| PLH020101 | CE | ✖ | 1 | 1 | 98 |
| PLH220022 | CE | ✖ | 1 | 1 | 100 |
