# Supplementary material for: Deficiencies in Natura 2000 for protecting recovering large carnivores: A spotlight on the wolf Canis lupus in Poland
Source: PLoS One. 2017 Sep 5;12(9):e0184144. doi: 10.1371/journal.pone.0184144 (PMC5584752; doi:10.1371/journal.pone.0184144)
Supplement: S2 Table — (DOCX) [file pone.0184144.s002.docx]

**Table 2. Statistical significance of the Mann-Whitney U-tests for the pairwise comparisons of protected habitat area sizes between wolf populations in Poland.**

| **Comparison** | **P-values** |
| --- | --- |
| **Baltic:Carpathian** | 0.437 |
| **Carpathian:CE** | 0.015 |
| **Baltic:CE** | 0.007 |
